# The Current Distribution of *Oncomelania hupensis* Snails in the People’s Republic of China Based on a Nationwide Survey

**DOI:** 10.3390/tropicalmed8020120

**Published:** 2023-02-14

**Authors:** Chao Lv, Yin-Long Li, Wang-Ping Deng, Zi-Ping Bao, Jing Xu, Shan Lv, Shi-Zhu Li, Xiao-Nong Zhou

**Affiliations:** 1 National Institute of Parasitic Diseases, China CDC (Chinese Center for Tropical Diseases Research), Key Laboratory on Parasite and Vector Biology, National Health Commission, WHO Collaborating Centre for Tropical Diseases, National Center for International Research on Tropical Diseases, Ministry of Science and Technology, Shanghai 200025, China; 2 School of Global Health, Chinese Center for Tropical Diseases Research, Shanghai Jiao Tong University School of Medicine, Shanghai 200025, China; 3 One Health Center, Shanghai Jiao Tong University, The University of Edinburgh, Shanghai 200025, China

**Keywords:** *Oncomelania hupensis*, schistosomiasis japonica, nationwide survey, existing snail habitat, area inhabited by snail

## Abstract

Schistosomiasis is a helminth infection caused by the genus *Schistosoma*, which is still a threat in tropical and sub-tropical areas. In the China, schistosomiasis caused by *Schistosoma japonicum* is mainly endemic to the Yangtze River valley. The amphibious snail *Oncomelania hupensis* (*O. hupensis*) is the unique intermediate host of *S. japonicum*; hence, snail control is a crucial approach in the process of schistosomiasis transmission control and elimination. In 2016, a nationwide snail survey was conducted involving all snail habitats recorded since 1950 in all endemic counties of 12 provinces. A total of 53,254 existing snail habitats (ESHs) were identified, presenting three clusters in Sichuan Basin, Dongting Lake, and Poyang Lake. The overall habitat area was 5.24 billion m^2^, of which 3.58 billion m^2^ were inhabited by *O. hupensis*. The area inhabited by snails (AIS) in Dongting and Poyang Lakes accounted for 76.53% of the population in the country. Three typical landscape types (marshland and lakes, mountains and hills, and plain water networks) existed in endemic areas, and marshland and lakes had a predominant share (3.38 billion m^2^) of the AIS. Among the 12 endemic provinces, Hunan had a share of nearly 50% of AIS, whereas Guangdong had no ESH. Ditches, dryland, paddy fields, marshland, and ponds are common habitat types of the ESH. Although the AIS of the marshland type accounted for 87.22% of the population in the whole country, ditches were the most common type (35,025 or 65.77%) of habitat. Six categories of vegetation for ESHs were identified. A total of 39,139 habitats were covered with weeds, accounting for 55.26% of the coverage of the area. Multiple vegetation types of snail habitats appeared in the 11 provinces, but one or two of these were mainly dominant. Systematic sampling showed that the presence of living snails was 17.88% among the 13.5 million sampling frames. The occurrence varied significantly by landscape, environment, and vegetation type. The median density of living snails in habitats was 0.50 per frame (0.33 m × 0.33 m), and the highest density was 40.01 per frame. Furthermore, two main clusters with high snail densities and spatial correlations indicated by hotspot analysis were identified: one in Hunan and Hubei, the other in Sichuan. This national survey is the first full-scale census on the distribution of *O. hupensis*, which is significant, as transmission interruption and elimination are truly becoming the immediate goal of schistosomiasis control in China. The study discerns the detailed geographic distribution of *O. hupensis* with the hotspots of snail density in China. It is beneficial to understand the status of the snail population in order to finally formulate further national control planning.

## 1. Introduction

Schistosomiasis is a helminth infection caused by blood flukes in *Schistosoma*, and it has intestinal or urogenital forms of clinical illness [1]. As the third most devastating tropical disease globally, schistosomiasis affects 240 million people worldwide and imperils more than 700 million people living in rural and poor settings in endemic countries, causing 1.4–3.3 million disability-adjusted life years (DALYs) annually [2,3,4]. Comfortingly, the global trends of morbidity and those infected show a declining pattern due to a series of integrated approaches, including persistent mass drug administration (MDA), improved water, sanitation, and hygiene (WASH), behavioral changes, and snail control [5,6]. Recently, the World Health Organization (WHO) launched a new set of guidelines on six evidenced-based recommendations to update the global public health strategy against schistosomiasis, and they have determined a more ambitious policy goal of eliminating schistosomiasis as a public health problem and moving towards the interruption of transmission worldwide [5,7].

Infection of *Schistosoma* is acquired after exposure to freshwater harboring cercariae released from intermediate host snails. Generally, there has been a strict consistency between the endemic areas of schistosomiasis and the geographical distribution of exclusive intermediate host snails [8,9,10]. Therefore, natural foci of six kinds of human schistosomiasis occur in dissimilar regions due to the unequal geographical distribution of the intermediate host snails.

Schistosomiasis caused by *Schistosome japonicum* (*S. japonicum*) is exclusively found in several East and Southeast Asian countries, including China, Japan, Indonesia, and the Philippines [11,12]. Schistosomiasis was formerly well known as a ruthless killer, the “God of Plague” in China, in which around 10 million patients were found in the 1950s. Schistosomiasis was prevalent in 12 provinces in the Yangtze River valley, involving 28,376 villages in 450 counties [13]. *Oncomelania hupensis* (*O. hupensis*), an amphibious snail species, is the unique intermediate host of *S. japonicum* in China. Consequently, snail control is considered to be an extremely effective and affordable approach based on the theory of interrupting the complete chain of schistosomiasis transmission [14,15]. Snail control through environmental modification and mollusciciding was once implemented as a core strategy between the 1950s and the 1980s, resulting in a rapid decline in the prevalence of schistosomiasis and even elimination of the disease in Shanghai, Zhejiang, Guangdong, Fujian, and Guangxi [9]. Nowadays, snail control is still an indispensable component of the new integrated strategy that was implemented after 2004, which focuses on infectious source control [9,16,17].

In 2015, schistosomiasis control in China achieved the criteria of transmission control, i.e., (1) the infection rate of humans and livestock was less than 1%; (2) there were no local acute schistosomiasis patients; (3) there were no *Schistosoma*-positive snails in two consecutive years, and schistosomiasis transmission interruption and elimination reasonably became the next step [18]. In 2016, the Plan of Health China 2030 issued by the State Council of China set a deadline on schistosomiasis elimination nationwide. However, the wide distribution of *O. hupensis* is one of the major challenges to schistosomiasis elimination, since the cryptic wildlife–snail interface may continuously exist [19,20]. Correspondingly, a habitat of area of 19,600 m^2^ inhabited by positive snails was detected, interrupting the record in which no positive snail was detected for 6 consecutive years starting in 2014 [21]. Subsequently, the geographical area inhabited by *O. hupensis* has remained at 3.6 billion m^2^ over fifteen years, although the snail control measure of large-scale molluscicide spraying was implemented every year. Such a phenomenon implies a tough spot in snail control. Indeed, the majority of the area inhabited by snails (AIS) was located in marshland in lake regions [21]. The application of molluscicide can effectively decrease snail density and hence lower the risk of schistosomiasis infection [22], but it does not work in marshlands of large size. Further, AIS and snail density are affected by floods, project construction, and landscape transplantation, causing AIS expansion and a snail emergency or re-emergency [23,24]. Therefore, the population dynamics and ecological characteristics of *O. hupensis* should be paid more attention, especially in the face of restricted use of molluscicides in the context of the national conservation program of Yangtze River [25].

The conventional way to record the distribution of *O. hupensis* at a detailed scale is to manually draw the habitats, which hinders a temporospatial analysis on the dynamics of habitats and hence the utilization of historical records in policy making. Fortunately, spatial information technology, e.g., geographic information systems (GIS) and global positioning systems (GPS), have powerful spatial data management and analysis capacities that offer spatial information on snail distribution and related environmental factors, which can then be visualized [26]. In addition, aside from mapping the spatial distribution of *O. hupensis* and its related environmental factors to master geographical distribution characteristics, various studies with respect to spatial correlations, clusters, predictions of snail breeding areas, and snail monitoring in part of an area have been carried out [27,28,29].

Although annual routine surveillance on snails has been conducted, the accurate mapping of *O. hupensis* at a national level has not yet been carried out. To gain a detailed national map of snail habitats and characterize their present distribution pattern, a nationwide survey on *O. hupensis* was conducted in 2016. It should be the most comprehensive snail survey nationwide so far, contributing to the ambitious goal of national schistosomiasis elimination and providing fundamental data for subsequent surveillance and research on snails.

## 2. Materials and Methods

### 2.1. Survey Area

A national survey on *O. hupensis* was conducted in 454 counties (administrative areas have changed in subsequent years) of 12 endemic provinces, representing the whole endemic area of schistosomiasis in China. All snail habitats recorded since 1950 were included as objects in the survey. A habitat was defined as a relatively isolated environment inhabited by *O. hupensis*. It could be a ditch, rice paddy, canal, pond, marshland, and so on.

### 2.2. Mapping Snail Habitats

National training for mapping habitats and field surveys was conducted among professional staff from provincial and county centers for schistosomiasis control (CSC). All habitats inhabited by *O. hupensis* recorded in archives (e.g., annual surveillance reports and databases) were reviewed by the skilled staff in local CSCs. All identified habitats were numbered using a unique code of 13 digits, of which the first 10 digits were the uniform and standardized country codes, representing a community, and the 11–13th digits were the codes of a specific habitat. The coordinates of key points of a habitat were determined by global positioning systems (GPS, Trimble GeoExplorer, Trimble Navigation Limited, Sunnyvale, CA, USA). A frame of the habitat was drawn based on the key point coordinates In Google Earth. Of note, habitats that are extinct due to changes in land use were also mapped by digitalizing paper maps in historical records. However, a further snail survey was unnecessary.

### 2.3. Field Survey on Snails

Two methods were employed in the field survey, namely, systematic sampling and environmental sampling (looking for snails only in suspected environments). The existing snail habitats (ESHs), in which *O. hupensis* snails were found in last two years before the present survey, were directly surveyed by systematic sampling. Environmental sampling was then used when no snails were found in the systematic sampling survey. The number of environmental sampling frames was not less than 20% of the sample size in previous instances of systematic sampling. Historical snail habitats were first surveyed by environmental sampling, and then by systematic sampling if snails were found in the habitats.

Implementation strategies of systematic sampling varied with different habitat sizes and shapes. Briefly, square frames of 0.33 m × 0.33 m were placed at equal intervals of 5 m or 10 m along linear environments such as ditches and canals or the periphery of polygonal environments such as ponds. Several parallel survey lines were set up every 5 m or 10 m in flat environments such as rice paddies and marshland, and sampling frames were placed on each survey line at intervals of 5 m or 10 m. Marshlands in Yangtze River or in major lakes normally have a huge range. When a marshland was less than 200,000 m^2^, systematic sampling was carried out at the intersection points between parallel horizontal and vertical lines at intervals of 20 m. If the area of marshland exceeded 200,000 m^2^, the intervals of the sampling lines could be 30 m or 50 m. In addition, the sampling size was no less than 500 frames.

All living *O. hupensis* snails that were identified by morphological features within the frames were collected and counted. The habitat types and vegetation types were recorded during the survey. The total area of the environments and the area inhabited by *O. hupensis* were estimated.

### 2.4. Data Management and Analysis

All collected data were input into Excel (Microsoft Office, v. 2016) to establish a database. The snail density of a habitat was defined as the number of living snails divided by the number of sampling frames. Area inhabited by snails (AIS) could include the partial range of a habitat, since a habitat was not necessarily occupied completely by *O. hupensis* snails. Correspondingly, the term “environmental area of snail habitat” (EASH), was used to describe the total area of a habitat. SPSS software (version 19.0, SPSS Inc., Chicago, IL, USA) was used for the corresponding descriptive analysis. The attribute data of habitats were linked to shape files by a unique 13-digit code. ArcGIS (version 10.0, ESRI, Redlands, CA, USA) was employed to visualize the distribution of habitats and for spatial analysis, e.g., hotspot analysis of snail density. The prevalence of *S. japonicum* in snails was not included in this dataset.

## 3. Results

### 3.1. Geographical Distribution of Existing Snail Habitats

A total of 53,254 ESHs were identified through the nationwide survey. The total EASH was 5.24 billion m^2^, of which 3.58 billion m^2^ were inhabited by *O. hupensis*. The ESHs were discontinuously distributed in the Yangtze River valley. Three clusters were found, namely, Sichuan Basin, Dongting Lake, and Poyang Lake.

**Figure 1:**
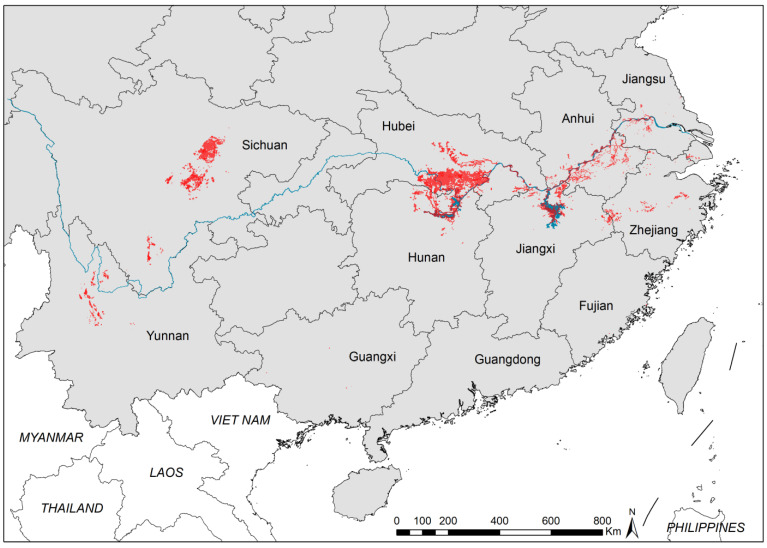
Distribution of existing snail habitats in China.

Although most of ESHs were located in the Yangtze River valley, the distribution patterns were different in the upper and lower reaches. ESHs were commonly found in smaller river systems in mountainous areas in the upper reaches, whereas they were mainly distributed in the marshlands along Yangtze River and its major branches in the middle and lower reaches (Figure 1).

**Figure 2:**
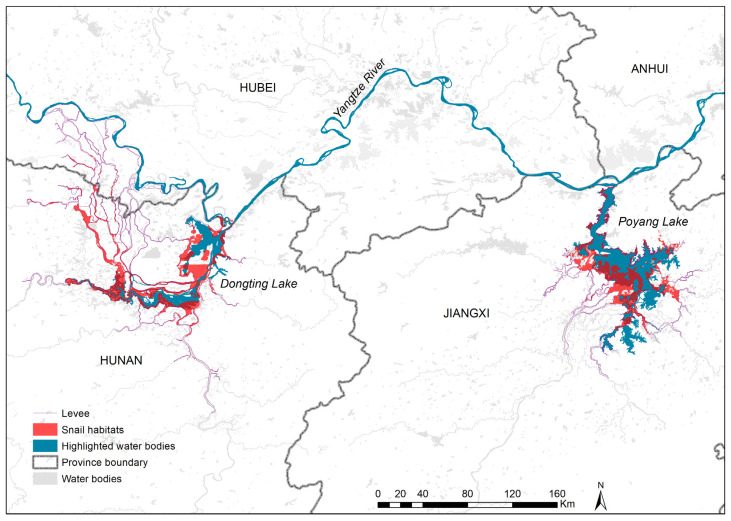
Distribution of existing snail habitats in Dongting and Poyang lakes.

The two biggest freshwater lakes (Dongting located in Hunan province and Poyang in Jiangxi province) in China harbored 76.53% of the AIS, although the corresponding number of habitats only accounted for 3.47% of the AIS in the whole country (Figure 2). The average AIS was 1.49 million m^2^ in the lake regions. The shares of AIS in Dongting and Poyang lakes were 50.65% and 25.88% of the total, respectively.

Three typical landscape types existed in schistosomiasis endemic areas, i.e., marshland and lakes, mountains and hills, and plain water networks. The AIS in these three types accounted for 3.38 billion m^2^, 0.19 billion m^2^, and 0.05 billion m^2^, respectively. Hunan province alone had a share of nearly 50% AIS in marshland and lakes, followed by Jiangxi and Hubei provinces. The other nine provinces had less than 10%. Sichuan (45.79 million m^2^, 23.79%), Hunan (41.54 million m^2^, 21.58%), and Anhui (35.31 million m^2^, 18.34%) provinces were home to the top three distributions of AIS in mountainous areas and hills. Snail habitats of plain water networks were almost all located in Jiangsu province, with 4.80 million m^2^. Other snail habitats of plain water networks were in Shanghai (0.034 million m^2^) and Zhejiang (0.008 million m^2^).

**Figure 3:**
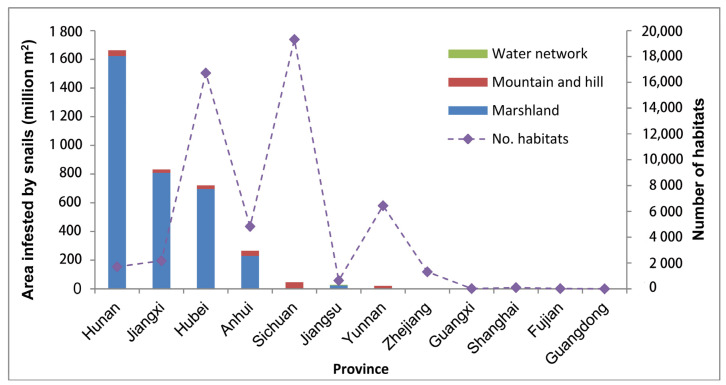
Area inhabited by snails in three landscape types and amount of existing snail habitats in 12 provinces.

Except for Guangdong, where no *O. hupensis* snails were found, Sichuan province had the largest number of 19,314 ESHs, followed by Hubei province, with 16,718 ESHs. Guangxi, with only 10 ESHs, had the fewest snail habitats among the 11 provinces. Hunan accounted for the largest proportion of EASH (1940.76 million m^2^ or 37.06%) and AIS (1663.64 million m^2^ or 46.51%), followed by Jiangxi (24.34%/23.27%) and Hubei (21.63%/20.16%). Shanghai possessed the least amount of EASH, as did Fujian for AIS (Table 1 and Figure 3).

### 3.2. Environmental Characteristics of Existing Snail Habitats

The common habitat types were ditches, dryland, paddy fields, marshland, and ponds. Although marshland types were only responsible for 10.17% of the surveyed area, the AIS in marshlands accounted for 87.22% of the area in the whole country (Table 2). In contrast, ditches were the most common type of habitat found (35,025 or 65.77%), but the AIS explained only 6.93% of the total.

**Figure 4:**
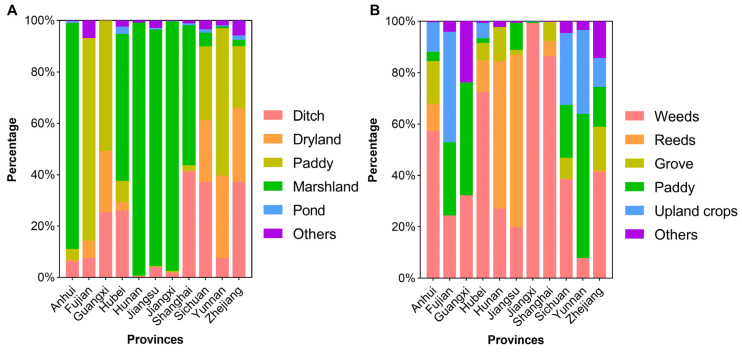
Composition of habitat types (**A**) and vegetation types (**B**) of existing snail habitats in 11 provinces. Guangdong was excluded due to its having no existing snail habitats by 2016.

Habitat types varied in different provinces. The dominant type was marshland in Jiangxi, Jiangsu, Hunan, and Anhui provinces and paddy fields in Fujian Province. The three provinces of Zhejiang, Sichuan, and Guangxi mainly included three types of environments of ditches, drylands, and paddy fields. There were mainly two environmental types of snail habitats in Yunnan Province, dryland and paddy fields, and the two main types in Shanghai were ditches and marshland (Figure 4A).

It was shown that ESH was covered with weeds, upland crops, reeds, groves, paddies, and other unclassified types of vegetation (Table 2). The ESH covered with weeds, paddies, and upland crops were numerous in quantity. A total of 39,139 (or 73.49%) weed-covered ESHs were identified, which accounted for 55.26% of AIS. Multiple vegetation types commonly appeared in 11 provinces, but only one or two types were dominant. Weeds occupied the largest area of snail habitats in the six provinces, i.e., Shanghai, Jiangxi, Hubei, Anhui, Zhejiang, and Sichuan. Besides weeds, groves and upland crops also held a high proportion of ESH in the aforementioned provinces. Reeds growing in marshland were the most important vegetation type in Jiangsu and Hunan province. Paddy was the most common type in Yunnan and Guangxi, whereas it was upland crops in Fujian (Figure 4B).

### 3.3. Incidence and Density of O. hupensis

Systematic sampling was performed in 51,905 out of 53,254 habitats. *O. hupensis* was discovered in 96.62% habitats. A total of 13.50 million sampling frames were set and examined for living *O. hupensis*. The overall presence of living snails among the sampling frames was 17.88%. The density of living snails was often low, although it could be as high as 40.01 per frame. The median density of living snails in habitats was only 0.50 per frame. As a supplementary survey, environmental sampling was conducted in 17,162 habitats, including 910 habitats in which systematic sampling was not performed. The occurrence of *O. hupensis* was recorded in 15,080 habitats. *O. hupensis* was found in 85,642 out of 2.76 million sampling frames.

**Figure 5:**
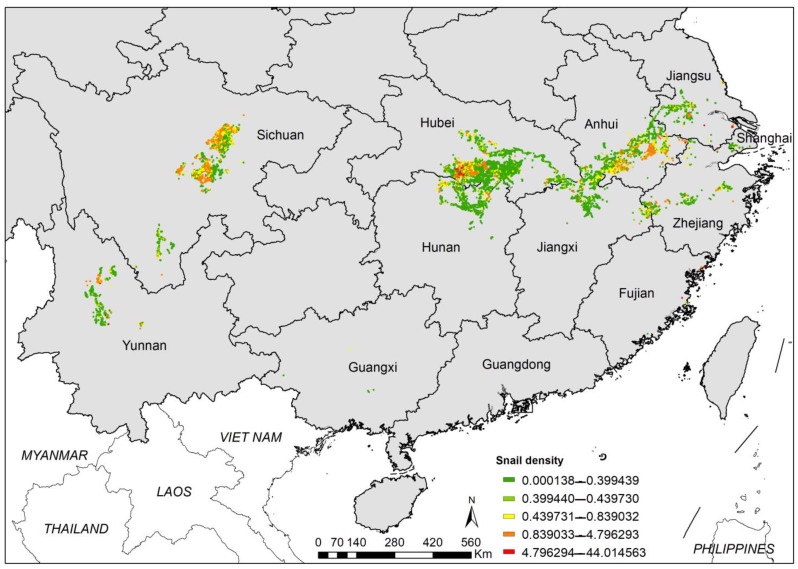
Density of living snails in exiting snail habitats.

The occurrence and density of sna”ls s’owed various patterns among landscapes. The highest occurrence (22.26%) and median density (0.51 per frame) were observed in mountainous and hilly areas (Table 3 and Figure 5). Significant variation was also seen in habitat and vegetation types (Table 2). Ditches with weeds and dryland with crops were the preferential habitats of *O. hupensis*.

**Figure 6:**
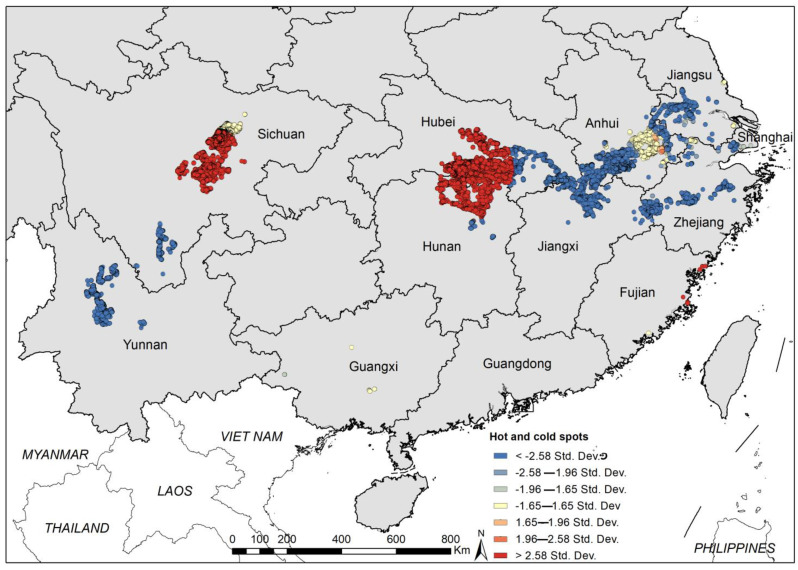
Hotspot analysis of snail density.

The snail density showed heterogeneity across the country. It was found that there were two main clusters with a high snail density and spatial correlation indicated by hotspot analysis, one in Hunan and Hubei, the other in Sichuan (Figure 6). On the other hand, lower snail densities were located in Yunnan, south of Sichuan, the middle-lower Yangtze River, and in Zhejiang mountainous regions.

## 4. Discussion

The national survey provided a high-resolution map of *O. hupensis* habitats for the first time and identified 53,254 ESHs over 3.58 billion m^2^ across the country. Approximately 90% of AIS was distributed in three provinces, i.e., Hunan, Jiangxi, and Hubei, located in the middle reach of the Yangtze River. The AIS in Dongting and Poyang lakes accounted for 76.53% of AIS. Therefore, these are the tough spots for schistosomiasis elimination in China. Indeed, Hunan and Jiangxi provinces have not yet achieved transmission interruption to date [30]. One of the core components of the current strategy for schistosomiasis control is snail control in the habitats that are frequently accessed by humans and domestic animals [31,32]. The implementation of molluscicide is not yet available in far-reaching habitats in the lakes. However, wild animals have been found that were infected with *S. japonicum* [33,34,35], which indicates that a natural life cycle of the parasite could be maintained between wild animals and *O. hupensis*. A sensitive surveillance and response system should be established for transmission among wild animals in far-reaching habitats, and One Health approaches should be introduced to prevent transmission from nature to humans.

In contrast to the marshland habitats in the bed of the Yangtze River and major branches and affiliated lakes, the majority of habitats in water network types in the Yangtze River delta were eliminated in the 1960s and the 1970s due to the development of agriculture and water conservancy [9]. The water network plain at that time consisted of Shanghai, southern Jiangsu, and northern Zhejiang. Only 423 ESHs in the region were identified in the present survey. According to historical records, over 125,000 habitats with a total AIS of 1.38 billion m^2^ were distributed in the delta [36]. It was estimated that over 5 million human cases occurred in the most heavily endemic region in China [37,38,39]. Now, the Yangtze River delta has become the most developed region, and high urbanization is consolidating the elimination of snail habitats. No local transmission has occurred since transmission interruption in Shanghai (1985), Zhejiang (1995), and Jiangsu (2019) [9].

Hubei province had an AIS of 721.11 million m^2^, which accounted for 20.16% of the AIS in the country. Of note, except for 1251 habitats located in the bed of Yangtze River and its major branches, 15,467 habitats consisting of 308.49 million m^2^ were distributed in irrigation plains, which was similar to the habitats in the Yangtze River delta. However, elimination of the snail habitats in the irrigation plains was not commonly achieved as in the delta, although the irrigation plains also witnessed the development of agriculture and water conservancy. The main reason for the lack of elimination is waterlog due to the maintainable higher water level of Yangtze River in the flooding season [40]. The proportion of eliminated habitats is 61.35% in the irrigation plain, which is far from the threshold of 99% of schistosomiasis elimination and maintenance that could be achieved [36]. In addition, given that humans and animals frequently access the ditch habitats, a high snail density in ditches is also a risk factor in schistosomiasis transmission.

In Sichuan and Yunnan provinces located in the upper stream of the Yangtze River, 25,754 ESHs were found, accounting for 48.36% of habitats in the country. Furthermore, the snail density in this region was also significantly higher than in others. However, the AIS was only 66.10 million m^2^ (or 1.85%). The snail habitats are often the small ditches around farmland either in plains or on terraces [41]. The transformation from rice paddies to dryland in some mountainous areas showed good effectiveness in snail control and schistosomiasis transmission interruption [42]. However, routine application of molluscicide is hindered, particularly in terraces, due to transportation problems and water sources. Roaming cattle are commonly observed in the schistosomiasis endemic areas of Yunnan and Sichuan provinces. The amount of cattle in the two provinces is responsible for over 50% of the population in all 450 endemic counties of 12 provinces [30]. Rodents play an important role in local transmission of schistosomiasis in mountainous areas [43,44]. Although transmission interruption has been achieved in humans and domestic animals, the wide distribution of *O. hupensis* and the high risk of its occurrence in wild animals could probably introduce schistosomiasis to humans.

A decrease of 98% in AIS was the criteria for transmission control of schistosomiasis between the 1970s and the 1990s. Indeed, five provinces, including Guangdong, Shanghai, Fujian, Guangxi and Zhejiang, eliminated over 99% of AIS when schistosomiasis elimination was achieved in the 1980s and the 1990s [36]. Local transmission of schistosomiasis has not occurred in these areas since the AIS was eliminated. Jiangsu province, which recently achieved transmission interruption, eliminated 98% of AIS by 2019 [36]. The proportion of eliminated AIS in the six other endemic provinces ranged from 47.11% to 84.53%. The present strategy of schistosomiasis control focuses on decreasing the sources of infection, e.g., ill humans and domestic animals, have facilitated schistosomiasis elimination. Meanwhile, a sensitive surveillance system for humans and domestic animals has been established and works well to facilitate the response to foci. Evidence has proved the effectiveness of this strategy [45]. However, schistosomiasis transmission among wild animals, not yet included in the surveillance system, might play an important role in the local transmission of schistosomiasis [43,44]. Given the difficulty in the management of infections among wild animals, decreasing the population of AIS is still a good choice for schistosomiasis elimination. In addition, sensitive surveillance and response systems to *Schistosoma*-positive snails should be applied. Novel technologies for the detection of cercariae in snails and the environment, e.g., loop-mediated isothermal amplification (LAMP) and bionic animal skin, should be assessed and used in these systems. Certainly, we also should pay more attention to the habitats with positive snails. Aside from general molluscicidal methods for the elimination of all snails in these habitats, the source of positive snails should be a focus. This scenario may be more in accordance with the requirements of environmental protection and will not cause species’ ecological imbalance. Achieving traceability of the sources of snail infection is not an easy job, as we still do not grasp the source of the re-emergence of positive snails in 2020 in Anhui [21]. Nowadays, One Health working at human–animal–environment interface may give us a new opportunity to clarify the complicated traceability problems.

The following suggestions are recommended for schistosomiasis elimination. First, nationwide planning of snail control measures should be formulated. The habitats with a high risk of schistosomiasis transmission should be identified, and these populations should be disposed of in a most reasonable method. Watershed ecological approaches can be used in planning [41,46]. Secondly, technological breakthroughs in snail control are needed. Conventional approaches to snail control, e.g., manual spraying of niclosamide, have shown low efficacy. Unmanned aerial vehicles, widely applied in agriculture, can outperform the human ability to spray molluscicide. Thirdly, risk assessment, including infection in wild animals, should be incorporated into the national surveillance system.

There were some shortcomings in the paper. First, the data used in the analysis were from a national survey conducted in 2016. Nevertheless, the results from the present analysis are still valuable for policy making, since the distribution of ESH and AIS has been very stable in the last decades [47]. Second, the national survey did not take into consideration the resurgence of *O. hupensis*. Therefore, the re-emerging habitats were unknown in the present survey. In fact, re-emergence has commonly been observed in recent years. For example, Guangdong province has maintained the same status of no *O. hupensis* presence for 38 years. However, *O. hupensis* were discovered at the boundary of two counties in 2019 shortly after the present nationwide survey [48]. The re-establishment of *O. hupensis* is a challenge to the achievement of schistosomiasis control and elimination.

## 5. Conclusions

Snail control is an indispensable measure in schistosomiasis control and elimination. The fundamental and essential challenge is to characterize the spatiotemporal distribution and dynamics of the snail population and thereafter to identify and control the factors of intermediate host snails. At the beginning of the elimination campaign, the results from the present nationwide survey will provide evidence for the formulation of national snail control planning measures. The database is expected to be updated dynamically in the future and should serve the precision control of schistosomiasis in the country.

## Figures and Tables

**Table 1:** Distribution of existing snail habitats and snail densities based on systematic sampling by province.

Province	No. ESH	EASH (Million m^2^)	Percentage of EASH (%)	AIS (Million m^2^)	Percentage of AIS (%)	No. Surveyed ESH	No. Systematic Sampling Frames	Percentage of Frames with Living Snails (%)	Maximum Snail Density (/0.11 m^2^)	Median Snail Density (/0.11 m^2^)
Sichuan	19,314	143.77	2.75	45.79	1.28	19,124	2,944,065	35.37	18.00	0.75
Hubei	16,718	1132.75	21.63	721.11	20.16	15,964	3,962,343	18.72	44.01	0.53
Yunnan	6440	44.24	0.84	20.31	0.57	6262	782,564	8.64	39.33	0.20
Anhui	4830	545.34	10.41	265.03	7.41	4769	1,192,716	14.27	20.56	0.53
Jiangxi	2175	1274.58	24.34	832.35	23.27	2033	1,530,114	9.43	6.50	0.25
Hunan	1707	1940.76	37.06	1663.64	46.51	1683	1,737,538	9.59	3.46	0.18
Zhejiang	1315	16.82	0.32	0.97	0.03	1315	952,078	3.37	24.44	0.21
Jiangsu	641	137.56	2.63	27.92	0.78	641	358,080	12.47	38.18	0.17
Shanghai	90	0.19	0.00	0.03	0.00	90	26,175	13.27	6.17	0.33
Fujian	14	0.22	0.00	0.02	0.00	14	11,084	6.11	16.25	2.09
Guangxi	10	0.20	0.00	0.06	0.00	10	6904	18.11	0.81	0.05
Guangdong	0	0.00	0.00	0.00	0.00	0	0	0.00	0.00	0.00
Total	53,254	5236.45	100.00	3577.25	100.00	51,905	13,503,661	17.88	44.01	0.50

ESH: existing snail habitat; EASH: environmental area of snail habitat; AIS: area inhabited by snail.

**Table 2:** Habitat and vegetation types of existing snail habitats.

Types	No. ESH	EASH (Million m^2^)	AIS (Million m^2^)	Percentage of AIS (%)	No. Surveyed ESH	No. Systematic Sampling Frames	Percentage of Frames with Living Snails (%)	Maximum Density of Snails (/0.11 m^2^)	Median Density of Snails (/0.11 m^2^)
Environment									
Ditch	35,025	448.47	247.79	6.93	34,397	6,299,097	24.85	44.01	0.57
Dryland	3568	123.00	49.14	1.37	3384	920,401	17.57	38.18	0.50
Paddy	6760	218.54	99.32	2.78	6521	1,570,437	16.88	39.33	0.47
Marshland	5414	4306.53	3120.21	87.22	5265	3,993,404	8.39	6.53	0.20
Pond	1019	75.95	23.90	0.67	892	175,288	11.24	13.50	0.35
Other	1468	63.97	36.88	1.03	1446	545,034	12.31	15.42	0.40
Total	53,254	5236.45	3577.25	100.00	51,905	13,503,661	17.88	44.01	0.50
Vegetation									
Weeds	39,139	2999.70	1976.83	55.26	38,111	8,871,150	20.09	44.01	0.53
Reeds	903	1360.40	1088.49	30.43	897	1,009,701	8.74	12.88	0.18
Grove	1369	493.57	319.48	8.93	1363	718,425	11.00	15.42	0.28
Paddy	5841	147.58	51.32	1.43	5737	1,317,745	15.82	39.33	0.49
Dryland crops	3465	165.14	95.25	2.66	3272	897,400	18.87	21.00	0.51
Other	2537	70.05	45.88	1.28	2525	689,240	12.55	16.67	0.40
Total	53,254	5236.45	3577.25	100.00	51,905	13,503,661	17.88	44.01	0.50

ESH: existing snail habitat; EASH: environmental area of snail habitat; AIS: area inhabited by snail.

**Table 3:** Snail densities in different landscape types.

Endemic Types	No. Surveyed ESH	No. Systematic Sampling Frames	Percentage of Frames with Living Snails (%)	Maximum Density of Snails (/0.11 m^2^)	Median Density of Snails (/0.11 m^2^)
Marshland and lakes	17,300	6,820,036	13.93	44.01	0.44
Mountains and hills	34,182	6,421,207	22.26	39.33	0.51
Plain water networks	423	262,418	13.17	38.18	0.21
Total	51,905	13,503,661	17.88	44.01	0.50

ESH: existing snail habitat.

## Data Availability

No additional data are available.

## References

[1] McManus D.P., Dunne D.W., Sacko M., Utzinger J., Vennervald B.J., Zhou X.N. (2018). Schistosomiasis. Nat. Rev. Dis. Primers..

[2] Colley D.G., Bustinduy A.L., Secor W.E., King C.H. (2014). Human schistosomiasis. Lancet.

[3] Murray C.J., Vos T., Lozano R., Naghavi M., Flaxman A.D., Michaud C., Ezzati M., Shibuya K., Salomon J.A., Abdalla S. (2012). Disability-adjusted life years (DALYs) for 291 diseases and injuries in 21 regions, 1990-2010: A systematic analysis for the Global Burden of Disease Study 2010. Lancet.

[4] GBD 2017 DALYs and HALE Collaborators (2018). Global, regional, and national disability-adjusted life-years (DALYs) for 359 diseases and injuries and healthy life expectancy (HALE) for 195 countries and territories, 1990–2017: A systematic analysis for the Global Burden of Disease Study 2017. Lancet.

[5] Lo N.C., Bezerra F.S.M., Colley D.G., Fleming F.M., Homeida M., Kabatereine N., Kabole F.M., King C.H., Mafe M.A., Midzi N. (2022). Review of 2022 WHO guidelines on the control and elimination of schistosomiasis. Lancet. Infect. Dis..

[6] Webster J.P., Molyneux D.H., Hotez P.J., Fenwick A. (2014). The contribution of mass drug administration to global health: Past, present and future. Philos. Trans. R. Soc. Lond. B. Biol. Sci..

[7] Ogongo P., Nyakundi R.K., Chege G.K., Ochola L. (2022). The Road to Elimination: Current State of Schistosomiasis Research and Progress Towards the End Game. Front. Immunol..

[8] Song L.G., Wu X.Y., Sacko M., Wu Z.D. (2016). History of schistosomiasis epidemiology, current status, and challenges in China: On the road to schistosomiasis elimination. Parasitol. Res..

[9] Xu J., Steinman P., Maybe D., Zhou X.N., Lv S., Li S.Z., Peeling R. (2016). Evolution of the National Schistosomiasis Control Programmes in The People’s Republic of China. Adv. Parasitol..

[10] Zhou X.N., Li S.Z., Hong Q.B., Yang K., Lv S., Xu J. (2018). Remain true to our original aspiration for farewell to the God of Plague, compose the new chapter for the national schistosomiasis control programme scientifically—Commemoration of 60th anniversary of publishing Chairman Mao Zedong’s two poems “Farewell to the God of Plague”. Chin. J. Schisto. Control.

[11] Chen J., Xu J., Bergquist R., Li S.Z., Zhou X.N. (2018). “Farewell to the God of Plague”: The Importance of Political Commitment Towards the Elimination of Schistosomiasis. Trop. Med. Infect. Dis..

[12] Gordon C.A., Kurscheid J., Williams G.M., Clements A.C.A., Li Y., Zhou X.N., Utzinger J., McManus D.P., Gray D.J. (2019). Asian Schistosomiasis: Current Status and Prospects for Control Leading to Elimination. Trop. Med. Infect. Dis..

[13] Zhou X.N., Bergquist R., Leonardo L., Yang G.J., Yang K., Sudomo M., Olveda R. (2010). Schistosomiasis japonica control and research needs. Adv. Parasitol..

[14] Zayed K.M., Guo Y.H., Lv S., Zhang Y., Zhou X.N. (2022). Molluscicidal and antioxidant activities of silver nanoparticles on the multi-species of snail intermediate hosts of schistosomiasis. PLoS. Negl. Trop. Dis..

[15] Muhsin M.A., Wang X., Kabole F.M., Zilabumba J., Yang K. (2022). The Indispensability of Snail Control for Accelerating Schistosomiasis Elimination: Evidence from Zanzibar. Trop. Med. Infect. Dis..

[16] Sun L.P., Wang W., Zuo Y.P., Hong Q.B., Du G.L., Ma Y.C., Wang J., Yang G.J., Zhu D.J., Liang Y.S. (2017). A multidisciplinary, integrated approach for the elimination of schistosomiasis: A longitudinal study in a historically hyper-endemic region in the lower reaches of the Yangtze River, China from 2005 to 2014. Infect. Dis. Poverty..

[17] Wang L.D., Chen H.G., Guo J.G., Zeng X.J., Hong X.L., Xiong J.J., Wu X.H., Wang X.H., Wang L.Y., Xia G. (2009). A strategy to control transmission of Schistosoma japonicum in China. N. Engl. J. Med..

[18] Lei Z.L., Zhou X.N. (2015). Eradication of schistosomiasis: A new target and a new task for the National Schistosomiasis Control Porgramme in the People’s Republic of China. Chin. J. Schisto. Control..

[19] Bergquist R., Zhou X.N., Rollinson D., Reinhard-Rupp J., Klohe K. (2017). Elimination of schistosomiasis: The tools required. Infect. Dis. Poverty..

[20] Xu J., Lv S., Cao C.L., Li S.Z., Zhou X.N. (2018). Progress and challenges of schistosomiasis elimination in China. Chin. J. Schisto. Control.

[21] Zhang L.J., Xu Z.M., Yang F., Dang H., Li Y.L., Lu S., Cao C.L., Xu J., Li S.Z., Zhou X.N. (2021). Endemic status of schistosomiasis in People’s Republic of China in 2020. Chin. J. Schisto. Control..

[22] Li Z.J., Ge J., Dai J.R., Wen L.Y., Lin D.D., Madsen H., Zhou X.N., Lv S. (2016). Biology and Control of Snail Intermediate Host of Schistosoma japonicum in The People’s Republic of China. Adv. Parasitol..

[23] Guo S.Y., Li L., Zhang L.J., Li Y.L., Li S.Z., Xu J. (2021). From the One Health Perspective: Schistosomiasis Japonica and Flooding. Pathogens.

[24] Li Y.S., Raso G., Zhao Z.Y., He Y.K., Ellis M.K., McManus D.P. (2007). Large water management projects and schistosomiasis control, Dongting Lake region, China. Emerg. Infect. Dis..

[25] Wang S.L., Li Y.L., Zhang L.J., Lu S., Xu J. (2019). Thinking on schistosomiasis control under the strategy of China’s Yangtze River Economic Belt. Chin. J. Schisto. Control.

[26] Kirby R.S., Delmelle E., Eberth J.M. (2017). Advances in spatial epidemiology and geographic information systems. Ann. Epidemiol..

[27] He J., Li W., Bergquist R., Zhang J.F., Shi L., Zhao S., Wu F., Yang K. (2016). The spatio-temporal distribution of *Oncomelania hupensis* along Yangtze river in Jiangsu Province, China after implementation of a new, integrated schistosomiasis control strategy. Geospat. Health.

[28] Hu F., Ge J., Lv S.B., Li Y.F., Li Z.J., Yuan M., Chen Z., Liu Y.M., Li Y.S., Ross A.G. (2019). Distribution pattern of the snail intermediate host of schistosomiasis japonica in the Poyang Lake region of China. Infect. Dis. Poverty.

[29] Niu Y., Li R., Qiu J., Xu X., Huang D., Shao Q., Cui Y. (2019). Identifying and Predicting the Geographical Distribution Patterns of Oncomelania hupensis. Int. J. Environ. Res. Public. Health.

[30] Zhang L.J., Xu Z.M., Yang F., He J.Y., Dang H., Li Y.L., Cao C.L., Xu J., Li S.Z., Zhou X.N. (2022). Progress of schistosomiasis control in People’s Republic of China in 2021. Chin. J. Schisto. Control.

[31] Wang X., Wang W., Wang P. (2017). Long-term effectiveness of the integrated schistosomiasis control strategy with emphasis on infectious source control in China: A 10-year evaluation from 2005 to 2014. Parasitol. Res..

[32] Cao Z.G., Zhao Y.E., Lee Willingham A., Wang T.P. (2016). Towards the Elimination of Schistosomiasis japonica through Control of the Disease in Domestic Animals in The People’s Republic of China: A Tale of over 60 Years. Adv. Parasitol..

[33] Liu X.P., Wang T.P., Wang Q.Z., Yin X.M., Zhou L., Wang F.F., Wang Y., Fang G.R. (2013). Infection status of sources of schistosomiasis japonica in marshaland and hill regions. J. Pathogen. Biol..

[34] Lv S.B., Chen N.G., Liu Y.M., Zhou L.Y., Wang Y.S., Hu F., Li Y.F., Yuan M., Lin D.D. (2019). Survey of Schistosoma japonicum infections in wild animals in hilly transmission-controlled areas of Jiangxi Province. Chin. J. Schisto. Control.

[35] Song J., Du C.H., Dong Y., Shen M.F., Zhang Z.Y., Bie S.S. (2022). Review on changes in major infectious sources and control effects of schistosomiasis japonica in hilly areas of Yunnan and Sichuan during different prevention and treatment stages. J. Med. Pest. Control.

[36] Zhou X.N. (2022). Report on National Survey of Oncomelania Hupensis in China.

[37] Zhou X.N. (2020). A high-quality driver to accelerate the progress towards schistosomiasis elimination by science and technology-led innovation in Jiangsu Province. Chin. J. Schisto. Control.

[38] Wen L.Y., Zhu D.M., Yan X.L., Chen J.H., Zhang J.F., Tao H.Q. (2007). Report on surveillance of schistosomiasis in Zhejiang province from1996 to 2005. Chin. J. Schisto. Control.

[39] Wang X.M. (1988). Review on the Progress of Schistosomiasis Elimination.

[40] Zhang S.Q., Sun C.S., Wang M., Lin D.D., Zhou X.N., Wang T.P. (2016). Epidemiological Features and Effectiveness of Schistosomiasis Control Programme in Lake and Marshland Region in The People’s Republic of China. Adv. Parasitol..

[41] Chen S., Lu D., Duan L., Ma B., Lv C., Li Y.L., Lu S.N., Li L.H., Xu L., Wu Z.S. (2022). Cross-watershed distribution pattern challenging the elimination of Oncomelania hupensis, the intermediate host of Schistosoma japonicum, in Sichuan province, China. Parasit. Vectors..

[42] Liu Y., Zhou Y.B., Li R.Z., Wan J.J., Yang Y., Qiu D.C., Zhong B. (2016). Epidemiological Features and Effectiveness of Schistosomiasis Control Programme in Mountainous and Hilly Region of The People’s Republic of China. Adv. Parasitol..

[43] Zou H.Y., Yu Q.F., Qiu C., Webster J.P., Lu D.B. (2020). Meta-analyses of Schistosoma japonicum infections in wild rodents across China over time indicates a potential challenge to the 2030 elimination targets. PLoS. Negl. Trop. Dis..

[44] VAN Dorssen C.F., Gordon C.A., Li Y., Williams G.M., Wang Y., Luo Z., Gobert G.N., You H., McManus D.P., Gray D.J. (2017). Rodents, goats and dogs—Their potential roles in the transmission of schistosomiasis in China. Parasitology.

[45] Zhang L.J., Li S.Z., Wen L.Y., Lin D.D., Abe E.M., Zhu R., Du Y., Lv S., Xu J., Webster B.L. (2016). The Establishment and Function of Schistosomiasis Surveillance System Towards Elimination in The People’s Republic of China. Adv. Parasitol..

[46] Chen S., Lv S. (2022). Watershed ecology-based thinking of *Oncomelania* snail control. Chin. J. Schisto. Control.

[47] Wang W., Bergquist R., King C.H., Yang K. (2021). Elimination of schistosomiasis in China: Current status and future prospects. PLoS. Negl. Trop. Dis..

[48] Huang S., Mao Q., Zhong Q., Fan X., Li W., Rao Y., Pei F., Li S., Deng Z. (2021). Reappearance of Risk of Schistosomiasis Transmission and the Response After 27 Years of Interrupted Transmission—Guangdong Province, China, 2019. China. CDC. Wkly..

